# Large-scale high-throughput screen for cardiac ryanodine receptor targeted therapeutics

**DOI:** 10.1016/j.jbc.2025.110951

**Published:** 2025-11-17

**Authors:** Roman Nikolaienko, Elisa Bovo, Jonathan C. Solberg, Marzena Brinkmann, Levy M. Treinen, Andrew R. Thompson, Kaja Berg, David D. Thomas, Jennifer J. Thomas, Donald M. Bers, Courtney C. Aldrich, Aleksey V. Zima, Razvan L. Cornea, Robyn T. Rebbeck

**Affiliations:** 1 Department of Cell and Molecular Physiology, Loyola University Chicago, Stritch School of Medicine, Maywood, Illinois, USA; 2 Department of Biochemistry, Molecular Biology, and Biophysics, University of Minnesota, Minneapolis, Minnesota, USA; 3 Department of Medicinal Chemistry, University of Minnesota, Minneapolis, Minnesota, USA; 4 Photonic Pharma LLC, Minneapolis, Minnesota, USA; 5 Department of Pharmacology, University of California at Davis, Davis, California, USA

**Keywords:** fluorescence resonance energy transfer (FRET), cardiomyocyte, calmodulin (CaM), calcium channel, calcium intracellular release, biosensor, calcium leak, drug screening, high-throughput screening (HTS), isoxazole

## Abstract

In high-throughput screening (HTS) assays using fluorescence lifetime (FLT)-detected FRET, we have identified compounds that allosterically modulate the pathologically leaky ryanodine receptor (RyR) calcium release channels. These compounds may prevent or reduce the elevated Ca^2+^ that fuels arrhythmia, heart failure, and age-related neurodegeneration. RyRs are responsible for intracellular Ca^2+^ release from endoplasmic/sarcoplasmic reticulum (ER/SR). The resulting [Ca^2+^] pulse is a signal for many cellular processes, whereas sustained elevated [Ca^2+^] is pathologic. Our FRET-based HTS detects the pathology-linked RyR leaky state by monitoring binding of the accessory protein calmodulin and the DPc10 peptide (corresponding to RyR2 residues 2460–2495) known to perturb interdomain interactions within RyR2. Under conditions mimicking a pathological state, we have screened a 50,000-compound chemical library to identify small-molecule modulators of RyR2 in cardiac SR membranes. This screen yielded 603 compounds that reproducibly altered FRET. Based on FRET response profiles that align with therapeutic potential, 83 of those most promising compounds were purchased and validated by FRET dose response evaluation. Focusing on ten chemical scaffolds that desirably increase A-CaM binding, six representative compounds reduced RyR2 activity as measured by [^3^H]ryanodine binding. Ca^2+^ dynamics in HEK293 cells expressing human RyR2 or in cardiomyocytes highlighted the isoxazole group of hits as potentially therapeutic by targeting the pathological RyR2 leak state.

Control of Ca^2+^ signaling is essential to healthy muscle and neuronal function. Important players in this signaling are the ryanodine receptor (RyR) intracellular channels, which are the Ca^2+^ gatekeepers of the endoplasmic reticulum (ER) and its muscle-specialized variant, the sarcoplasmic reticulum (SR). Of the three mammalian isoforms, RyR2 is the dominant isoform expressed in the heart, and the main isoform expressed in the brain. Brain also expresses RyR1 (dominant isoform in skeletal muscle) and RyR3 (1, 2).

Pharmaceutically, RyRs are increasingly attractive for therapeutic discovery for treating dysfunctional ER/SR Ca^2+^ leak, which is prevalent in age-related pathologies, including arrhythmia, heart failure, sarcopenia, Alzheimer’s disease and Huntington’s disease (3, 4, 5, 6, 7, 8). Proof of principle for targeting RyR has been demonstrated by studies investigating RyR inhibitors that mitigate RyR Ca^2+^ leak in these pathologies (dantrolene (9, 10, 11, 12, 13, 14, 15, 16, 17), rycals (6, 18, 19, 20), carvedilol (21, 22, 23, 24)). Thus, there is a strong scientific basis for using systematic screening approaches to find new agents that inhibit ER/SR Ca^2+^ leak. Indeed, this has proven effective for identifying new compounds with therapeutic potential, particularly using HEK293 cells co-expressing RyR and ER Ca^2+^ indicator R-CEPIA1er (25, 26, 27, 28, 29, 30, 31). This screening approach has yielded exciting new compounds, despite being of a moderate throughput.

We have previously validated structure-based HTS assays for identifying RyR1- and RyR2-targeted modulators from small-compound libraries (32, 33, 34). Our RyR-targeted high-throughput screening (HTS) platform is designed to find both allosteric activators and inhibitors (not blockers) of RyR. Specifically, we measure binding of RyR regulators, FKBP12.6, calmodulin (CaM), and for RyR2 a peptide that disrupts inter-domain interactions (DPc10). We previously established that binding of CaM and DPc10 is negativity correlated and indicative of the functional state of RyR2 in SR membrane and cardiomyocyte preparations (32, 35, 36): the hyperactive leaky state of RyR2 has reduced CaM affinity and increased DPc10 binding (36, 37, 38, 39), and compounds that increase CaM binding and/or decrease DPc10 binding can reduce this pathological state (32, 35, 36). Dantrolene is a good example of an allosteric regulator that inhibits RyR activity, reduces DPc10 binding and increases CaM binding (35, 36, 39). This is despite dantrolene binding at a site away from the RyR pore and DPc10 and CaM binding sites (40).

Our HTS-compatible assay measures fluorescence lifetime (FLT; τ) to calculate FRET from an excited donor fluorophore attached to a single-Cys on FKBP12.6 (D-FKBP) and an acceptor fluorophore attached to a single-Cys on CaM (A-CaM) or the N-terminus of DPc10 peptide (A-DPc10), where these labeled proteins both bind to RyR2 in SR membranes (Fig. 1*A*). Shifts in FRET positively correlate with shifts in protein/peptide binding, though FRET is also sensitive to compounds (*e.g.* tacrolimus) that reduce FKBP12.6 binding (32). Based on binding sites on the RyR homotetramer, multiple acceptors could be within FRET range (10 nm) of the donors. However, for A-CaM FRET, we have shown that the contribution of acceptors on neighboring protomers to the FRET signal is negligible (41). Based on the higher resolution RyR2 cryo-EM, the RyR2 sequence corresponding to DPc10 is more centrally located between the positions of FKBP on one protomer and the neighboring protomer, and thus the FRET signal may have a contribution from A-DPc10 on the neighboring protomer (38).

**Figure 1:**
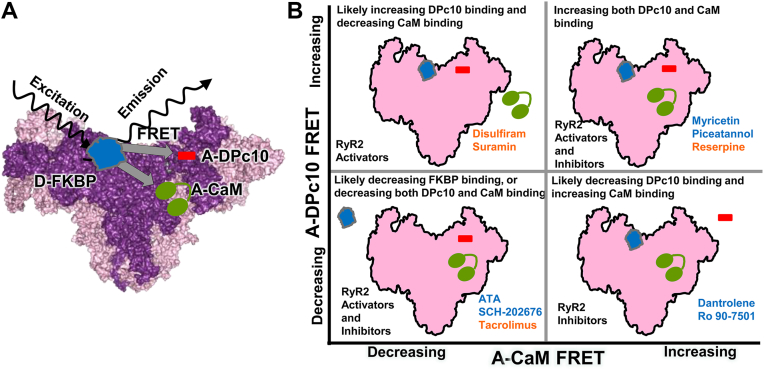
**HTS-compatible, FRET-based assay sensitive to binding of RyR2 modulators CaM, DPc10 and FKBP.***A*, schematic illustration of FKBP12.6 (*blue*), CaM (*green*) and peptide DPc10 (*red*) on RyR (32). The FRET assay involves preloading SR membranes (isolated from pig skeletal or cardiac muscle) with AF488-FKBP12.6 (D-FKBP), and removing unbound D-FKBP *via* centrifugation. After addition of sub-saturating concentrations of AF568-CaM (A-CaM) or HL647-DPc10 (A-DPc10), assay samples are loaded on 1536-well plates, and fluorescence decay is acquired to measure FRET. *B*, based on pilot screens, outcomes from A-CaM and A-DPc10 FRET readouts in response to modulators that increase (*orange*) or decrease (*blue*) RyR2 activity at nM Ca^2+^ (32, 34, 36).

The relationship between compound effects on FRET using A-CaM vs A-DPc10, and the functional effect on RyR2 at nanomolar free Ca^2+^ is illustrated in (Fig. 1*B*). Hit compounds identified by increasing A-DPc10 FRET and decreasing A-CaM FRET have typically been RyR2 activators. In contrast, Hit compounds that decrease A-DPc10 and increase A-CaM FRET are typically RyR2 inhibitors. These compounds are likely to be allosteric regulators, binding in a position that structurally alters both DPc10 and CaM binding.

For Hit compounds that decrease both FRET readouts, it is less clear which functional effect may be predominant. However, compounds that reduce FKBP12.6 binding, *e.g.* tacrolimus will inherently reduce both A-DPc10 and A-CaM FRET. Of note, both activators and inhibitors have been found in the quadrants where compound effects are in the same direction for FRET readouts. For compounds that increase both A-CaM and A-DPc10 FRET, it is possible that the CaM effect is functionally predominant. It is also unlikely that D-FKBP binding is increased in these cases, because unbound D-FKBP is removed during assay preparation, and the bound D-FKBP remains high during the lifetime of the experiment (<5 h) (42). Overall, in the majority of situations, FRET readouts reflect binding of A-DPc10 and A-CaM binding.

Here, we used our FRET assay to screen a 50,000-compound library for RyR1 and RyR2 modulators, using isolated skeletal and cardiac SR preparations, respectively, in diastolic conditions. Using *in vitro* functional assays, we subsequently identified a chemical structural class, isoxazole, with desirable inhibitory effects on RyR2 at resting conditions. This group of compounds also reduced RyR2-mediated ER Ca^2+^ leak in HEK293 cells, and increased SR Ca^2+^ load and Ca^2+^ transients in adult cardiomyocytes.

## Results

### HTS of a 50K-compound CNS-compatible compound library

To identify drug-like RyR inhibitors that may be compatible with development as leads for either cardiac or neurodegenerative diseases, we used our established RyR-CaM FRET assays (32, 34) to screen the 50K-compound ChemBridge CNS library. This library is composed of a diverse subset of ChemBridge’s larger stock of lead-like and drug-like small molecule compounds with a high probability of blood-brain barrier penetration and improved safety profiles. Furthermore, this library has been manually culled to remove common pan assay interference compounds. While the library contains structural analogs for structure-activity relationship (SAR) studies, additional analogs are available from ChemBridge’s 450K compound resources, which facilitates further testing of hit analogs in the process of lead development.

We have previously established our CaM-RyR FRET assay for screening a pilot library in 1536-well plates, with a focus on RyR using skeletal and cardiac SR (32, 34). Importantly, using native SR membranes isolated from porcine skeletal muscle or left ventricle allows for isoform comparison of RyR1 vs RyR2, respectively. Relative to human RyR1 and RyR2, the sequence identity of porcine orthologs is 96.7% and 98.1%, respectively.

The compound library was pre-loaded over forty 1536-well assay plates. Each plate had the following wells allocation: 1280 wells for 5 nl of library compounds (10 mM in DMSO for 10 μM final concentration), 224 wells for 5 nl DMSO, and 32 wells for 5 nl of suramin (20 mM in DMSO for 20 μM final concentration), a control that releases CaM from RyR. 60 minutes prior to loading, the FRET assay samples were prepared by incubating D-FKBP-labeled skeletal or cardiac SR with 150 or 100 nM A-CaM, respectively. The use of sub-saturating concentrations was designed to enable sensitivity for compounds that increased or decreased acceptor-labelled protein binding to RyR (43). Final assay conditions also included 30 nM Ca^2+^ to represent resting cellular [Ca^2+^]. To promote homogeneous populations of pathological RyR state associated with oxidative stress, cardiac SR was pre-treated with 100 μM H_2_O_2_ prior to labeling with fluorescent proteins, and skeletal SR FRET assay conditions included oxidized glutathione, as previously used in pilot screening (32, 34).

With two replicates per isoform assay, we acquired both FLT waveforms and fluorescence spectra, as previously described (33, 44, 45). Our previous studies with pilot screens demonstrated that the majority of reproducible Hits (identified in at least two of three screen replicates) were RyR functional modulators (32, 34).

**Figure 2:**
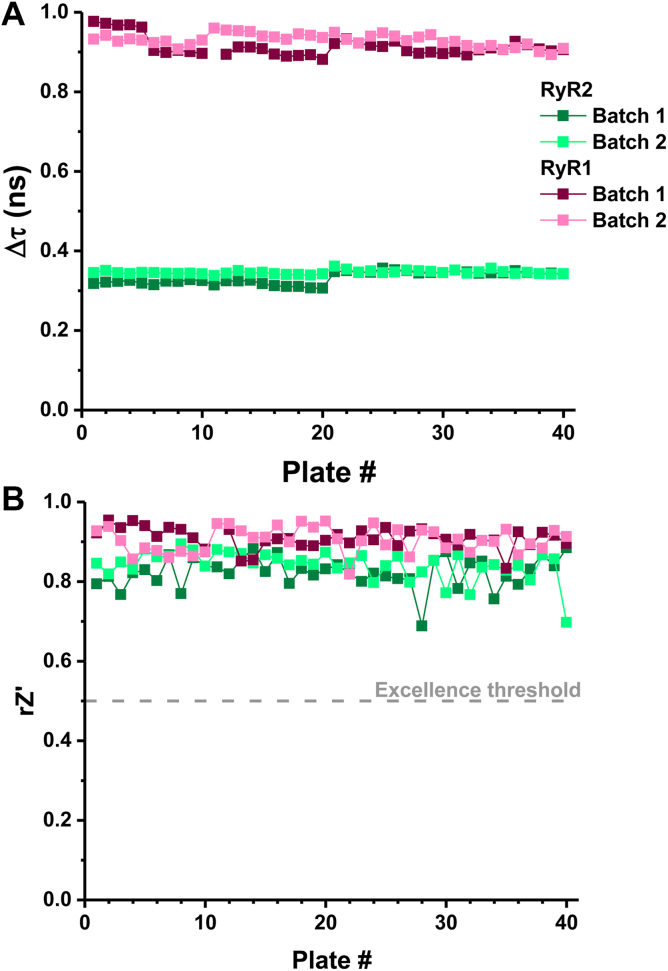
**Excellent performance of FRET assays across plates and runs.** Assay samples for FRET between D-FKBP and A-CaM bound to RyR1 (skeletal SR; *pink*) or RyR2 (cardiac SR; *green*) were loaded in duplicate (batches) onto 1536-well plates preloaded with CNS compound library, DMSO and a column of 20 μM suramin. Suramin is well established to strip CaM from RyR. Comparison between DMSO (negative control) and suramin (positive control) was used to assess assay performance, quantified by consistency between plates and high (*A*) Δτ and (*B*) robust (r) Z′.

To quantify how robust our CNS screens were across plates, we tested 20 μM suramin, a tool compound that is well-established to strip CaM-RyR1 binding (46, 47). This tool compound was loaded over column 48 of each library assay plate. Assay quality was high, as the large effect of suramin (relative to DMSO control) was consistent between preparations and replicates with an increase in FLT values (Δτ) from DMSO control values of 922 ± 16 ps and 339 ± 13 ps for RyR1 and RyR2 preparations, respectively (Fig. 2*A*). Furthermore, we demonstrate excellent rZ′ values throughout the screen, with values of 0.90 ± 0.03 and 0.83 ± 0.04 for RyR1 and RyR2 preparations, respectively (Fig. 2*B*). The rZʹ value uses statistical effect size and signal variation as a gauge of HTS assay quality, with 0.5 ≤ rZ′ < 1 indicating an excellent assay (48, 49).

**Figure 3:**
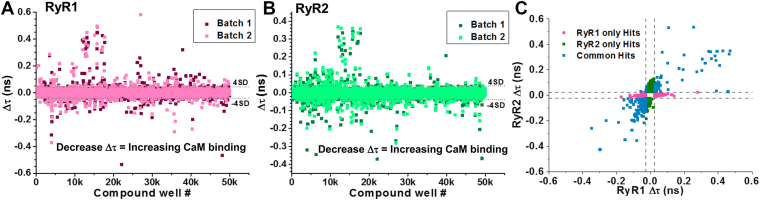
**HTS of 50K-compound CNS library for RyR modulators.** Assay samples for FRET between D-FKBP and A-CaM bound to RyR1 (skeletal SR; *pink*) or RyR2 (cardiac SR; *green*) were loaded in duplicate (batches) onto 1536-well plates preloaded with CNS library compounds, DMSO and a column of 20 μM suramin. Effects on FLT (Δτ) are displayed for RyR1 (*A*) and RyR2 (*B*) for each batch tested. Compounds that shift τ by more than 4 standard deviations of the mean are classified as Hits. Hits that increase CaM binding (indicated by lowering τ) are desirable, as displayed in Figure 1. *C*, orthogonal displays of the averaged effect of Hit compounds on RyR1 vs RyR2 FLT. Compound effects are categorized between RyR1 specific (*pink*), RyR2 (*green*), and isoform non-specific (*blue*).

Interfering compounds were removed based on previously established criteria of similarity index from fluorescence spectra (44, 45). For RyR1, we identified 647 (1.3% of the total library) and 764 (1.5%) compounds in experimental repeats (batches) 1 and 2, respectively, altered FLT by our threshold of four standard deviations (calculated from median of absolute deviation with correction by multiplication of 1.4826) from the plate-wide median (Fig. 3*A*). Between the two batches, 400 compounds were reproducibly altered Δτ past this threshold. Reproducible compounds past this threshold were termed “Hits”. For RyR2, although 748 hits were identified in each batch, 312 compounds were reproducible (Fig. 3*B*). Between the two isoforms, we identified 603 reproducible Hit compounds, with 18% overlapping between RyR1 and RyR2 screens (Fig. 3*C*).

Following the primary screen, these 603 compounds were selected for follow-up confirmation FRET assays. Using identical RyR-CaM FRET conditions as described above, each plate set contained three compound concentrations (10, 30 and 50 μM) over 3 wells each per compound. Compounds were taken from the library master plate. Further, these plates were also tested on assay conditions without the presence of A-CaM, named D-only. Of the 603 compounds, 261 undesirable compounds were removed on the basis that they had the same effect on D-only and D/A, thus likely quenching the donor, and/or did not alter D/A FLT by > 4SD. Based on previous reports, compounds that increase A-CaM binding, indicated by a reduction in Δτ value, were found to typically reduce RyR1/2 activity. Of the remaining 342 compounds, we prioritized the 83 compounds that increased A-CaM in RyR1 and/or RyR2. For all 83 prioritized Hits, fresh powder stocks were then purchased for follow-up FRET dose–response assays.

### FRET dose–response assay

**Figure 4:**
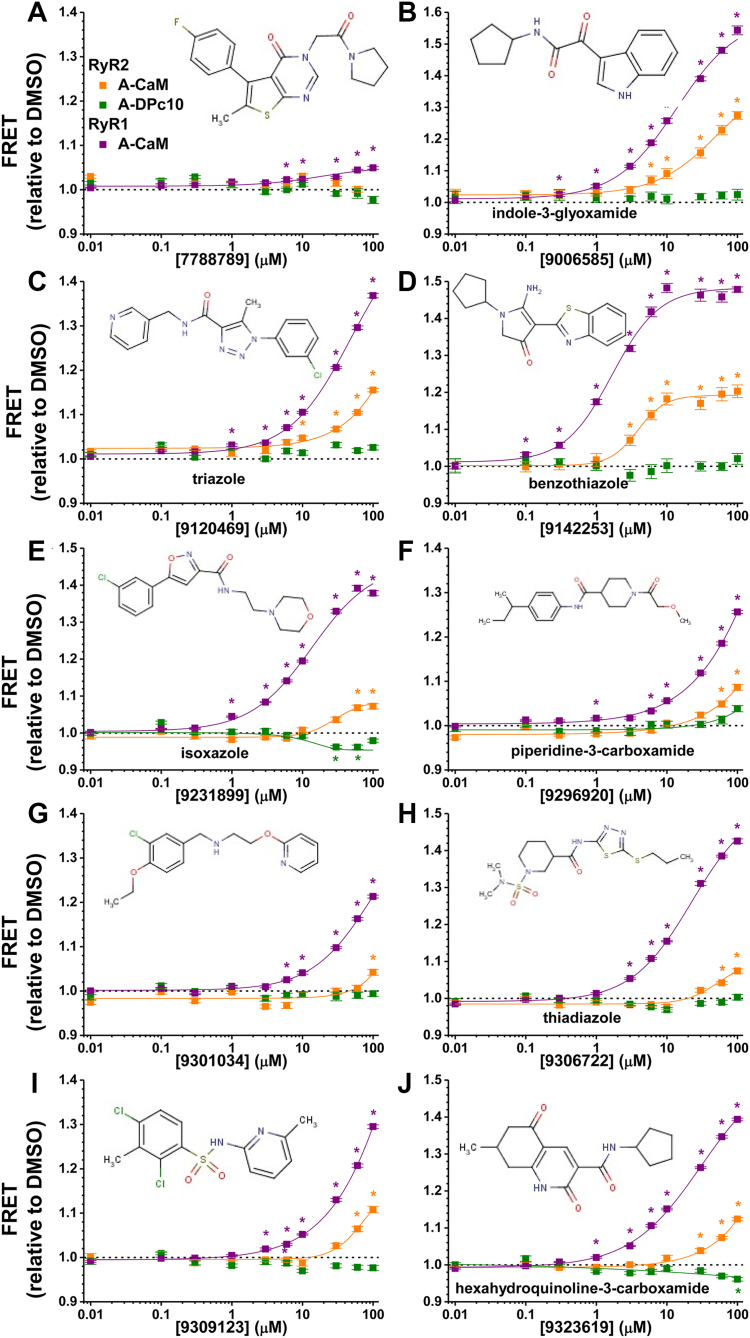
**FRET dose response for Hits representative of each chemical cluster.** The effect of each ChemBridge compound on FRET between D-FKBP and A-CaM on RyR1 (*purple*), A-CaM on RyR2 (*orange*) and A-DPc10 on RyR2 (*green*). FRET dose response representatives of (*A*) chemically unique 7788789, (*B*) indole-3-glyoxamide, (*C*) triazole, (*D*) benzothiazole, (*E*) isoxazole, (*F*) piperidine-3-carboxamide, (*G*) chemically unique 9301034, (*H*) thiadiazole, (*I*) chemically unique 9309123, and (*J*) hexahydroquinoline-3-carboxamide. Data shown as mean ± SD, n = 3. ∗Significance from DMSO control, *p* < 0.05, using unpaired, two way Student’s *t* test.

The selected Hits were further validated by first acquiring the FRET response to a range of Hit concentrations (0.01–100 μM) under the same assay conditions as the compound retest. In addition, we tested using our D-FKBP and A-DPc10 assay conditions with our cardiac SR preparation. As previously reported (32), this assay measures DPc10 binding, which was positively correlated with the leak state of RyR2 and reduced CaM binding (37). Of the selected Hits, 72 could be physicochemically grouped within one of 11 chemical clusters, and 11 additional Hits feature unique chemical scaffolds. The chemical structures are shown in the dose-response profiles, which are grouped by unique chemical structure (Fig. S1) or chemical cluster (Figs. S2–S7). In Figure 4, we show the effects of representative FRET modulators for each cluster or unique chemical structure that displays the desirable increase in A-CaM FRET with RyR2 and/or RyR1. In comparing the compound effect on the primary screen and the re-test, 79 compounds (out of 83 purchased) reproducibly altered FRET in a similar manner. The 1-benzothiophene-2-carboxamide, benzamide, N-arylpiperazine and N-aryl urea did not progress further due to undesirable effects, such as no effect on A-CaM FRET or increasing A-DPc10 FRET (Figs. S2, *A* and *B* and S6, *A* and *B*). The benzothiazole and indole-3-glyoxamide Hits all increased A-CaM FRET with RyR1 and RyR2, with no effects on A-DPc10 FRET (Figs. 4, *B* and *D*, S2*C*, and S3). Similarly, four of five triazole Hits increased CaM binding to both RyR1 and RyR2, with no effect on DPc10 binding (Figs. 4*C* and S7*B*). Hexahydroquinoline-3-carboxamide Hits increased CaM binding for both RyR1 and RyR2 for three of four compounds, with two compounds also reducing DPc10 binding (Figs. 4*J* and S2*D*). Similarly, only half of the piperidine-3-carboxamides increased CaM binding in RyR2, with one Hit slightly increasing DPc10 binding (Figs. 4*F* and S6*C*). Of the 30 isoxazole Hits shown in Figures 4*E*, S4, and S5, most increase A-CaM FRET for both RyR1 and RyR2, with 8 compounds having no effect on A-CaM FRET with RyR2, and two compounds reducing binding of A-CaM FRET with RyR2. Desirably, nine compounds that increased A-CaM FRET with RyR2, also decreased A-DPc10 FRET (Figs. 4*E*, S4, and S5). For the thiadiazole Hits, four of the six Hits increased A-CaM FRET with both RyR1 and RyR2, with half of those Hits also slightly increasing A-DPc10 FRET to a similar level of A-CaM FRET being increased (Fig. S7*A*). The representative Hits (Fig. 4) and a selection of isoxazole Hits progressed to functional testing using our radioligand binding assay ([^3^H]ryanodine binding).

### Effect of hits on RyR activity

As a medium-throughput assay, we first evaluated the functional impact of compounds using [^3^H]ryanodine binding assays, which yields an index of RyR activity in SR membranes (50). Similar to our FRET assays, pathology was induced by the presence of 100 μM H_2_O_2_ for cardiac SR and 5 mM GSSG for skeletal SR. To evaluate RyR function in conditions representing resting vs contraction Ca^2+^, experiments were undertaken with 0.1 or 30 μM Ca^2+^, respectively.

**Figure 5:**
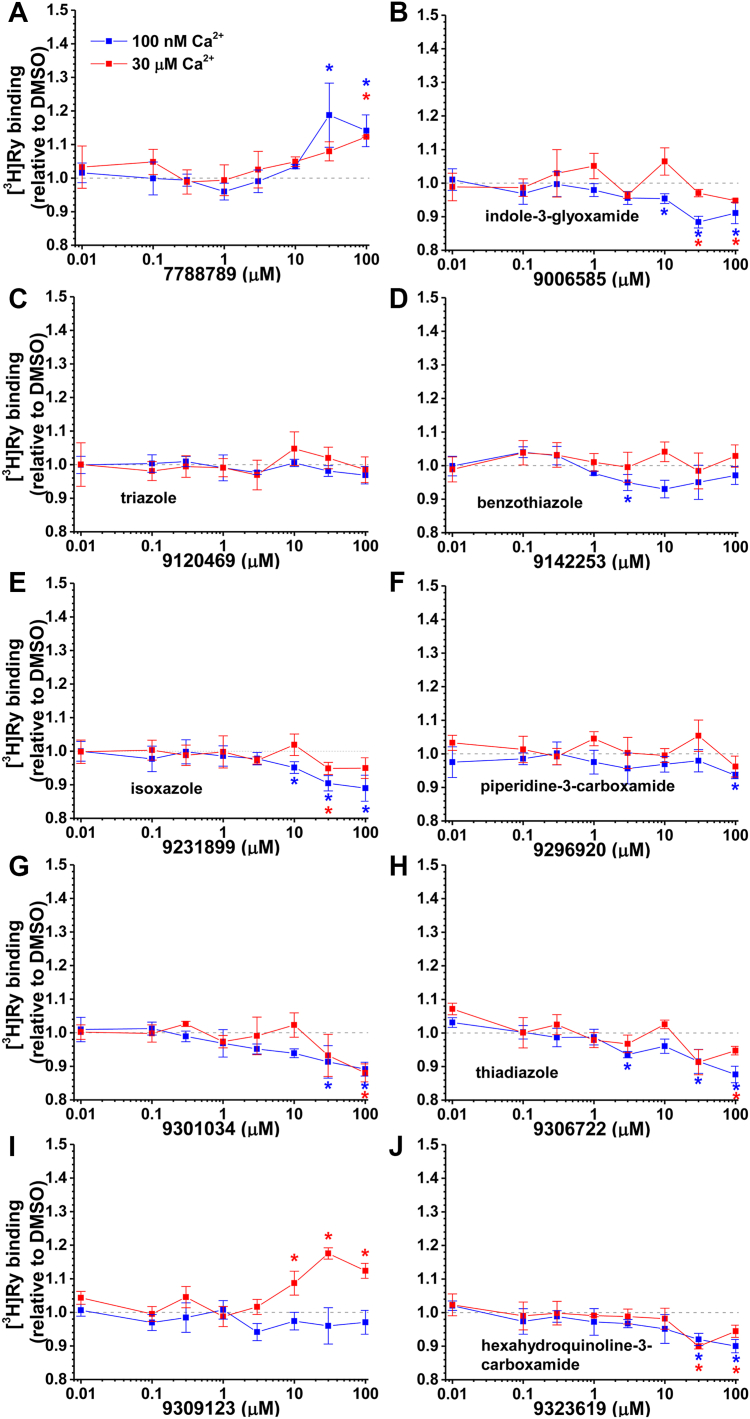
**[^3^H]ryanodine binding profiles for RyR2 in the presence of cluster-representative Hit compounds.** Dose-dependent (0–100 μM) effect of compounds on [^3^H]ryanodine binding to cardiac SR (RyR2) at 100 nM (*blue*) or 30 μM (*red*) free Ca^2+^. Dose response representatives of (*A*) chemically unique 7788789, (*B*) indole-3-glyoxamide, (*C*) triazole, (*D*) benzothiazole, (*E*) isoxazole, (*F*) piperidine-3-carboxamide, (*G*) chemically unique 9301034, (*H*) thiadiazole, (*I*) chemically unique 9309123, and (*J*) hexahydroquinoline-3-carboxamide. Data are shown relative to DMSO control, means ± SD, n = 3. ∗*p* < 0.05 for samples vs. DMSO control using Student’s two-way, unpaired *t* test.

As shown in Figure 5, seven of the ten representative compounds desirably reduced RyR2 activity at 0.1 μM Ca^2+^, which aligned with these compounds increasing CaM binding, and two of these compounds (isoxazole, 9231899; and hexahydroquinoline-3-carboxamide, 9323619) that had also reduced A-DPc10 FRET (Fig. 4). As exceptions to the inverse relationship between activity and FRET, compounds 9120469 and 9309123 did not alter RyR2 activity at 0.1 μM Ca^2+^. As an example of a compound that only altered A-CaM FRET with RyR1, not RyR2, it is interesting to note that 7788789 did alter RyR2 activity, though as a channel activator at both 0.1 and 30 μM Ca^2+^. On RyR1, all 10 representative Hits increased activity at 0.1 μM Ca^2+^, although the effects of the triazole (9120469) and the isoxazole (9231899) are relatively subtle (Fig. S8).

With a host of isoxazole compounds on hand, we functionally tested 10 analogs (including 9231899) on RyR1/2 function. Eight of these Hits significantly reduced RyR2 activity at 0.1 μM Ca^2+^ (Fig. S9). Seven of these compounds increased A-CaM FRET with RyR2 (Figs. S4 and S5), with 9205412 decreasing A-CaM FRET RyR2 (Fig. S4). One of the Hits (9226734) that did not alter RyR2 activity also had no effect on A-CaM FRET with RyR2, but did reduce DPc10 binding (Figs. S4 and S9*E*). For RyR1, all isoxazole compounds increased A-CaM FRET and increased RyR1 activity, though to varying levels, at 0.1 μM Ca^2+^ (Figs. S4, S5, and S9).

### Effect of Hit on ER Ca^2+^ in T-Rex-293 SERCA2a stable cell line expressing human RyR2

**Figure 6:**
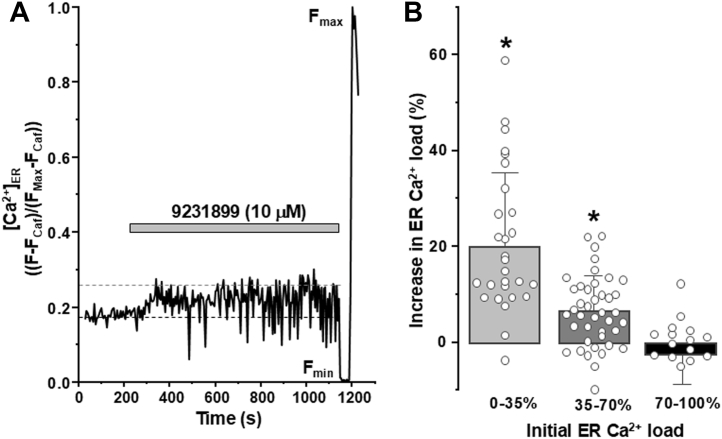
**Effect of a representative isoxazole (9231899) on ER Ca^2+^ load in a T-Rex-293 SERCA2a stable cell line expressing human RyR2 and SERCA2a.***A*, effect of 9231899 (10 μM) on [Ca^2+^]_ER_ in one representative T-Rex-293 SERCA2a stable cell line, as measured using ER Ca^2+^ sensitive protein R-CEPIA1er. Caffeine (10 mM) and ionomycin (2 μM) in 10 mM Ca^2+^ were used to normalize the signal (F_min_ and F_max_, respectively). *B*, averaged effects of 9231899 on relative increase in ER Ca^2+^ load compared with low (0–35%, n = 28 cells), moderate (35–70%, n = 41 cells) and high (70–100%, n = 19 cells) initial ER Ca^2+^ load. 9231899 increased ER Ca^2+^ load in cells with low, not moderate or high, initial ER Ca^2+^ load. Data shown as mean ± SD, individual cell data points shown in graph. ∗*p* < 0.05 *versus* initial ER Ca^2+^ load, using paired two-way Student’s *t* test.

To confirm the stabilizing effect of a representative isoxazole on human RyR2 in a cellular setting, we used an established ER Ca^2+^ dynamics assay in a T-Rex-293 SERCA2a stable cell line (51, 52). Cells were also transfected with human RyR2 and ER Ca^2+^ sensor, R-CEPIA1er. As a routine, cells were permeabilized to control the cytosolic environment, and ionomycin was used to calibrate the [Ca^2+^]_ER_ from R-CEPIA1er fluorescence. Due to differences in RyR2 expression level, the resting [Ca^2+^]_ER_ levels differed between cells (53). To account for this, the modulator effect is compared to the initial ER Ca^2+^ load, with low initial ER Ca^2+^ load associated with high RyR2 expression, and high initial ER Ca^2+^ load associated with low RyR2 expression. As shown in Figure 6, addition of representative isoxazole, 9231899 10 μM, increased ER Ca^2+^ load. This increase was the largest (20.2% ± 15.5%) and significant in the cells with lower initial ER Ca^2+^ load, which aligns with cells that likely have the greatest expression of RyR2. Indeed, the effect is lower (6.75% ± 7.34%) and null (−2.30 ± 7.33%) in cells that have moderate and high initial ER Ca^2+^ load, respectively, which aligns with these cells likely expressing little or no RyR2. With all cells likely expressing similar levels of SERCA2a, it is likely that 9231899 has little/no effect on SERCA2a activity. Thus, the effect of this isoxazole compound appears to be specific to cells expressing greater levels of functional RyR2, with little effect on ER Ca^2+^ load in cells with little/no RyR2 activity.

### Effect of hit on Ca^2+^ dynamics in cardiomyocytes

**Figure 7:**
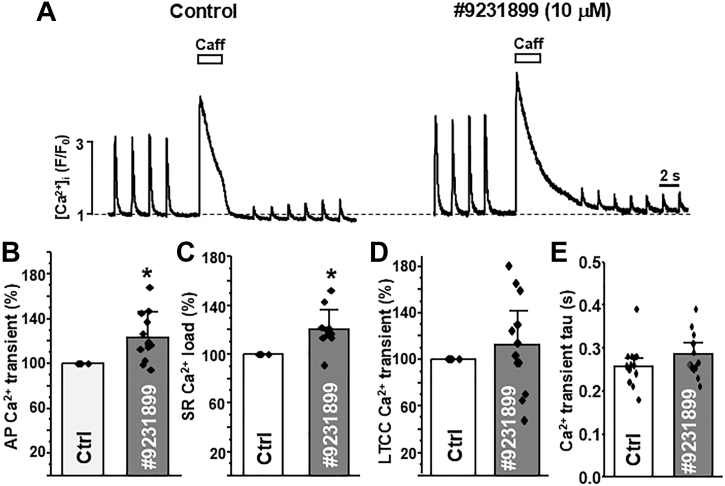
**Effect of representative isoxazole (9231899) on Ca^2+^ signaling in cardiomyocytes.***A*, representative F/F0 profiles for before (control; *left panel*) and after addition of 10 μM 9231899 (*right panel*) on Ca^2+^ transients in ventricular cardiomyocytes. From these line scans, the effect of 9231899 was calculated on the AP-induced Ca transient amplitude (*B*), caffeine-induced SR Ca^2+^ load (*C*), LTCC-mediated Ca^2+^ transient amplitude (*D*), and Ca^2+^ transient decay tau (*E*). Data shown as mean ± SD, individual data points shown (n = 12 cells). ∗*p* < 0.05 *versus* control, using paired two-way Student’s *t* test.

Using mouse ventricular cardiomyocytes, we assessed the effect of our representative isoxazole, 9231899, on the Ca^2+^ dynamics involved in action potential (AP)- and caffeine-induced transients. These Ca^2+^ transients were acquired during electrical field stimulation at 0.5 Hz before and after 5 min of perfusion with 10 μM isoxazole 9231899. The amplitude of the Ca^2+^ transient during caffeine addition was used as an index of SR Ca^2+^ load, and the first AP-induced Ca^2+^ transient after caffeine treatment (when there is little to no Ca^2+^ available for release) was used as a measure of Ca^2+^ influx *via* L-type Ca^2+^ channels (LTCC). Figure 7, *A* and *B* show that 9231899 increased AP-induced Ca^2+^ transient amplitudes by 22.9% ± 21.2%. Furthermore, we observe a 19.9 ± 16.2% increase in ER Ca^2+^ load (Fig. 7*C*) with 9231899. In contrast, the LTCC-mediated transients are not significantly altered by 9231899 (Fig. 7*D*), indicating that the isoxazole compound does not alter LTCC function. Additionally, 9231899 did not alter AP-induced Ca^2+^ transient decay tau (Fig. 7*E*), which in murine cardiomyocytes, is an index of SERCA2a function (7). Therefore, the increase in AP-induced Ca^2+^ transients is likely due to the enhanced SR Ca^2+^ load and release, but not to increases in either LTCC or SERCA function. Indeed, the increased SR Ca^2+^ load with 9231899 is most likely due to reduced SR Ca^2+^ leak *via* RyR2.

## Discussion

This study uses previously developed RyR biosensors to screen a 50K-compound library for drug-like small molecules that target RyR2 dysfunction, as early-stage screening for therapeutics for heart disease and neurodegeneration. For HTS, we used FRET-based assays that monitor CaM binding affinity to RyR1 and RyR2, based on the association between CaM binding to RyR2 and the healthy, non-leaky state of RyR2 in SR membrane preparations and cardiomyocytes. By screening the ChemBridge 50K CNS library, we identified drug-like, small molecules that could be progressed for lead development. The functional effects of Hits that likely increased CaM binding to RyR1/2 were validated using [^3^H]ryanodine binding assays, which confirmed several chemical groups as potential inhibitors of RyR2. Progressing to HEK293 cells recombinantly expressing human RyR2, we demonstrate that a representative isoxazole compound increases ER Ca^2+^ in cells with relatively low ER Ca^2+^. Similarly, this compound also increases SR Ca^2+^ and Ca^2+^ transient amplitude when tested on murine adult cardiomyocytes.

Similar to our validation of these RyR-CaM biosensors (32, 34), the screening performance was excellent with rZ′ values > 0.6 for every plate. By screening the library twice, we were able to progress with the Hits most likely to have an effect in the following assays. Moderate reproducibility was likely in part due to those compounds that were on the cusp of the threshold criteria in one screen batch and below the threshold criteria in the other screen batch. For this study, we chose to prioritize the reproducible Hits that increased CaM binding to RyR1/2 in the cherry-picked plates. Non-reproducible Hits or Hits that decreased CaM binding were considered less likely to reduce RyR2 leak, and so were de-prioritized.

From our FRET dose-response data, no Hits were identified as RyR2-selective. Indeed, many of the Hits had stronger effects on RyR1. The greater Δτ seen with RyR1 samples allows for the potential to detect more subtle RyR1 FRET modulators. Although RyR2 isoform selectivity would have been desirable, the lack of it is not viewed as an issue as future analog testing during lead development may yield selective compounds. This future lead development will be informed by the analogs also identified in this screen.

Prior validation of the RyR1-CaM biosensor using a pilot library (33, 34) indicated a negative correlation between FRET and RyR1 activity (using [3H]ryanodine binding), with the majority of Hits reducing FRET and increasing RyR1 activity. This also held with chloroxine and myericetin, which both increased CaM-FRET and reduced RyR1 activity (33, 34) in skeletal muscle fibers or with SR membranes (using [3H]ryanodine binding). This was curious at the time, as CaM is known to promote RyR1 opening at nanomolar Ca^2+^ in bilayers, but reduce opening at micromolar Ca^2+^ (54, 55). This contrasts to RyR2, for which CaM inhibits activity at all relevant [Ca^2+^] (56, 57). Upon testing a larger compound library, we found that the majority of Hits (18 of 19) that were functionally tested here increased RyR1-CaM FRET and decreased RyR1 activity at nM Ca^2+^. This FRET vs function relationship is inverted relative to the pilot screens but does align with CaM’s known function as an RyR1 activator in nM [Ca^2+^]. Future studies that focus on RyR1 inhibitors will involve studying the range of compounds that reduce A-CaM FRET with RyR1, with minimal/no effect on RyR2 FRET. Of note, for isoxazole compound 9231899, although a strong effect on CaM-RyR1 FRET was observed, only a subtle increase in RyR1 activity was observed by the highest concentration (100 μM) tested.

We found that 13 of 16 RyR2-CaM FRET increasers decreased RyR2 activity in [^3^H]ryanodine binding assays. This association aligns well with our previous data and understanding that CaM binding quiets RyR2. CaM is well-established as a RyR2 inhibitor, and binding has been associated with a non-leaky state of the RyR2 in cardiomyocytes. This has been most effectively demonstrated with dantrolene, which restores CaM binding and ameliorates RyR2 leak in cardiomyocytes (32, 35, 36). Similar to our pilot screen, we observe a pattern between DPc10 binding and RyR2 function, as seven (isoxazoles and hexahydroquinoline-3-carboxamide) of the RyR2 inhibitors that increased RyR2-CaM binding also decreased DPc10 binding. Accessibility of the DPc10 binding site is dependent on relaxed interaction between RyR N-terminus and helical domain 1 (38). Reduced DPc10 binding indicates that these domains are likely “zipped” to promote a closed RyR state. Our findings support at least two readouts of allosteric stabilizations of the RyR2 closed state. Isoxazole and hexahydroquinoline-3-carboxamide compounds promote a state that both enhances the availability of the CaM binding site and promotes inter-domain interactions between helical domain 1 and N-terminal domains, resulting in reduced DPc10 binding. In contrast, indole-3-glyoxamide and benzothiazole enhance CaM binding site availability without promoting inter-domain interactions that impact DPc10 binding. Representative piperidine-3-carboxamide and thiadiazole compounds also enhance CaM binding with no impact on DPc10 binding, but both have analogs that increase DPc10. With the different structural impacts, these compounds could be effective tools to resolve structural nuances from the many factors that can cause RyR2 structural states associated with pathologies (*e.g.* HF, CPVT).

Direct measurements of [Ca^2+^]_ER_ dynamics in T-Rex-293 SERCA2a stable cell line expressing human RyR2 revealed that isoxazole 9231899 increases ER Ca^2+^ load, particularly in cells with deleted Ca^2+^ load due to high RyR2 expression level and thus increased Ca^2+^ leak (Fig. 6). Analysis of intracellular Ca^2+^ dynamics in ventricular myocytes revealed that isoxazole 9231899 increases the SR Ca^2+^ load and AP-induced cytosolic Ca^2+^ transient amplitude (Fig. 7). Isoxazole 9231899, however, did not change LTCC-mediated Ca^2+^ transients, nor SERCA2a-mediaded Ca^2+^ transient tau. These results suggest that by partially inhibiting (not blocking) resting SR Ca^2+^ leak and increasing SR Ca^2+^ load, isoxazole 9231899 can enhance SR Ca^2+^ release and heart contraction during the action potential.

As isoxazoles (1) do not block RyR2 and (2) allosterically alter CaM and DPc10 binding, it is highly likely that isoxazoles bind to the cytoplasmic portion of the channel complex, away from the channel pore. Similar to isoxazoles, dantrolene increases CaM binding and decreases DPc10 binding (32, 35, 36). Therefore, possible binding sites could include the P1 phosphorylation domain, where dantrolene was shown to bind to RyR1 (40). The binding of CaM and DPc10 is also allosterically impacted by CPVT-associated mutants in the cytoplasmic domains, RyR2 phosphorylation and oxidation (35, 36, 39). Thus, it is difficult to assign a binding site on RyR2 at this time. The binding site could be confirmed in future studies using cryo-EM or drug docking (incorporating the range of data from analogs).

It is rare for a high-throughput screen to directly produce a drug compound. Usually, the Hits are used in lead compound development. From this screen, we have identified several compounds that would be promising for further lead development. Based on the functional assays, these compounds are isoxazoles (9231899), hexahydroquinoline-3-carboxamide (9323619), indole-3-glyoxamide (9006585), 9309123, and thiadiazole (9306722). The integration of an isoxazole ring can offer improved physicochemical properties, potentially improved drug efficacy, and pharmacokinetic properties, and often exhibits low toxicity. Because of unique structural characteristics, isoxazole interact noncovalently with various target enzymes and receptors by forming hydrogen bonds, π-π stacking and hydrophilic interactions. Isoxazole derivatives have a wide spectrum of therapeutic activities such as antimicrobial, antibacterial, antifungal, anti-inflammatory, anticancer, anti-viral, cardiovascular (the permeability transition pore inhibitors) and antidiabetic properties (58, 59, 60). Hexahydroquinoline is a well-known scaffold for developing new physiologically active compounds due to its synthetic flexibility. Hexahydroquinoline derivatives display various biological activities, such as anti-inflammatory, antifungal, and promising anticancer properties (61). Introducing glyoxamide provides a greater degree of versatility to the system with respect to a simple amide due to the possibility to form an additional hydrogen bond through the keto group and the variable glyoxamide torsional angle. The combination of the indole with the glyoxamides creates an excellent template, that is suitable for structural modifications either to produce a specific therapeutic effect or to improve the pharmacokinetic profiles of biologically active compounds (62). Thiadiazoles are capable of crossing the cellular membrane due to their mesoionic nature, and their good liposolubility is attributed to the presence of a sulfur atom. Although thiadiazoles have advantages over other commonly found therapeutic scaffolds, their toxicity remains a major concern (63). Pharmacologically, thiadiazole derivatives have shown antiprotozoal, antidiabetic, antioxidant, and anticancer activities (64).

This study highlights several compounds of interest for immediate future work. The RyR2 inhibitors can progress to analog testing for lead compound development. With FRET and [^3^H]ryanodine binding data for several isoxazole compounds, we are primed for structure-activity relationship studies to identify a more potent RyR2 inhibitor. In addition to further validation on Ca^2+^ dynamics in pathological cardiomyocyte models, future studies are necessary to determine effects on non-cardiac tissue, especially skeletal muscle, considering the effect most of these compounds have on RyR1 FRET and [^3^H]ryanodine binding. Concurrent with functional evaluation, additional studies will involve determining lead compatibility by measuring absorption, distribution, metabolism, excretion, and toxicity properties.

In addition to lead development, many of the Hits are of interest as tools for further evaluation of RyR structural regulation and CaM-RyR binding, particularly funneling into further testing with our developed assays to gauge structural dynamics of CaM regulation of RyR1/2 (41).

Overall, we have identified several new RyR2 inhibitors that are primed for further testing in disease models and hit-to-lead development.

## Experimental procedures

### Compound handling and preparation of 1536-well assay plates

The 50K ChemBridge CNS library compounds were received in 96-well plates, and reformatted into 1536-well flat, black-bottom polypropylene plates (Greiner Bio-One). Primary screening assay plates were prepared by transferring 5 nl of 10 mM compound stocks, control compound (suramin) or DMSO from 1536-well source plates to 1536-well black polypropylene plates using an Echo 550 acoustic dispenser. Columns 1 to 4 and 45 to 47 were loaded with DMSO only (compound-free controls), and column 48 was loaded with suramin (20 μM final). A complete set of the library was loaded in columns 5 to 44 over forty 1536-well plates. These assay plates were then heat-sealed using a PlateLoc Thermal Microplate Sealer (Agilent Technologies) and stored at −20 °C.

Cherry-pick assay plates were prepared by transferring 5, 15 or 25 nl of 10 mM compound stocks, control compound or DMSO from 1536-well source plates to 1536-well black polypropylene plates using an Echo 550 acoustic dispenser. Compound wells were back loaded with DMSO for total volume of 25 nl. Repurchased hit compounds for dose-response assays were dissolved in d_6_-DMSO for 10 mM stocks. Then, each compound was serially diluted in 96-well polypropylene plates, transferred to 384-well Echo-qualified compound plates then transferred to 1536-well assay plates using the Echo 550 acoustic dispenser. All assay plates were stored at −20 °C prior to usage. Before screening, compound plates were equilibrated to room temperature (22 °C).

### Sequence similarity and isolation of SR vesicles

Crude sarcoplasmic reticulum (CSR) vesicles were isolated from porcine *longissimus dorsi* muscle and porcine cardiac left ventricle tissue by differential centrifugation of homogenized tissue (56). Skeletal heavy SR vesicles, which are enriched in RyR1, were isolated by fractionation of crude skeletal SR vesicles using a discontinuous sucrose gradient (56). All vesicles were flash-frozen and stored at −80 °C. Immediately prior to the fluorescence or [^3^H]ryanodine binding studies described below, the SR vesicles were stripped of residual endogenous CaM by incubation with a peptide derived from the CaM binding domain of myosin light chain kinase, followed by sedimentation (65).

The protein sequence identity was calculated after alignment using UniProt ClustalO (66). For RyR1, the human (P21817.3) and porcine (P16960.2) sequences have 96.7% identity. For RyR2, the human (Q92736.3) and porcine (XP_020928342.1) sequences have 98.1% identity.

### Expression, purification and labelling of D-FKBP and A-CaM, and preparation of HL647-DPc10

Single-cysteine mutants of FKBP12.6 (C22A/T85C/C76I) and CaM (T34C) were expressed in *E. coli* BL21(DE3) pLysS (Agilent Technologies), purified, and respectively labeled, *via* thiol-maleimide reaction, with fluorescent probes AF488 or AF568, for D-FKBP and A-CaM, as described previously (50, 67). DPc10 peptides were synthesized and N-terminally labelled with HiLyte Fluor 647 (for A-DPc10) by AnaSpec corresponding to the human RyR2 sequence 2459-GFCPDHKAAMVLFLDRVYGIEVQDFLLHLLEVGFLP-2494.

### Preparation of SR vesicles for FRET measurements

Cardiac SR membranes were treated with 100 μM H_2_O_2_, labelled with D-FKBP, and FRET assay samples were prepared with 30 nM Ca^2+^ and 100 nM A-CaM or 1.5 μM A-DPc10 as previously described (32). Skeletal SR membranes were labeled with D-FKBP, and FRET assay samples were prepared with 150 μM A-CaM, 30 nM free Ca^2+^ and 5 mM GSSG as previously described (34). Solutions from all FRET assays included 20 mM PIPES, 150 mM KCl, 1 μg/ml aprotinin, 1 μg/ml leupeptin, 0.1 mg/ml BSA. Each SR FRET sample (2 mg/ml cardiac SR and 0.5 mg/ml skeletal SR) was loaded to the assay plates in aliquots of 5 μl/well using a Multidrop Combi reagent dispenser (Thermo Fisher Scientific) with a low-volume dispensing cassette (Thermo Scientific). For D-FKBP only SR samples, A-CaM or A-DPc10 was omitted.

### Fluorescence data acquisition

At 60 min after sample loading, fluorescence measurements were performed using high-throughput fluorescence plate-readers (Fluorescence Innovations) provided by Photonic Pharma LLC, including one detecting FLT and another detecting fluorescence spectra, as described previously (32, 34, 44, 45, 68, 69, 70, 71).

### HTS data analysis

Time-resolved fluorescence waveforms for each well were fitted based on a one-exponential decay function, using least-squares minimization global-analysis software, as detailed previously (45).

The FRET efficiency (*E*) was determined as the fractional decrease of donor FLT (τ_D_), due to the presence of acceptor fluorophore (τ_DA_), using the following equation:

(1) $$E=1-\frac{\tau_{DA}}{\tau_{D}}$$

HTS quality for the FRET assay per plate was confirmed using wells preloaded with control (DMSO) and tested tool compound as indexed by the Z′ factor:

(2) $${rZ}^{\prime }=1-3\frac{\left({{MAD}_{DMSO}*1.4826)+(MAD}_{Tool}*1.4826\right)}{\left|M_{DMSO}-M_{Tool}\right|}$$

where MAD_DMSO_ and MAD_Tool_ are the median of absolute deviation of the DMSO τ_DA_ and tool compound τ_DA_, respectively; M_DMSO_ and M_Tool_ are the medians of the DMSO τ_DA_ and tool compound τ_DA_, respectively.

Typically, the statistics-based criterion for Hit selection is that it changes the result by > 3SD. This is because the probability of a Hit being part of the normally-distributed control population is very small <0.3%, minimizing the probability of selecting false Hits. Based on pilot screens with these biosensors (32, 34), we know that an optimal threshold for categorizing a compound as a “Hit” is if the compound changes the τ_DA_ by > 4 SD (calculated by MAD multiplied by 1.4826) relative to control τ_DA_ (sample exposed to 0.1% DMSO).

### [^3^H]Ryanodine binding to SR vesicles

[^3^H]ryanodine binding to cardiac SR was undertaken using 96-well plates, as previously described (32), with the minor exception that 3 mg/ml, not 2 mg/ml, of SR was used in assay. In brief, final assay conditions contained 1% DMSO control or 0.01 to 100 μM [Hit], with 100 μM H_2_O_2_, and 7.5 nM [^3^H]ryanodine in *binding assay* media containing: 150 mM KCl, 1 μg/ml Aprotinin/Leupeptin, 1 mM EGTA, and 0.24 or 1.62 mM CaCl2 (as determined by MaxChelator to yield 100 nM or 30 μM of free Ca^2+^, respectively), 0.1 mg/ml BSA, 100 nM CaM, 5 mM sodium-adenosine triphosphate, and 20 mM PIPES (pH 7.0).

[^3^H]ryanodine binding to skeletal SR was undertaken using 96-well plates, as previously described (34). In brief, final assay conditions contained 1% DMSO control or 0.01 to 100 μM [Hit], with 100 μM H_2_O_2_, and 10 nM [^3^H]ryanodine in *binding assay* media.

Non-specific and maximal [^3^H]ryanodine binding to SR were separately assessed by addition of 40 μM non-radioactively labeled ryanodine or 5 mM adenylyl-imidodiphosphate and 20 mM caffeine, respectively. Such control samples were each distributed over 4 wells/plate. Binding of [^3^H]ryanodine was determined after a 3-h incubation (37 °C) and filtration through grade GF/B Glass Microfiber filters using a 96-channel Brandel Harvester. In 4 ml of Ecolite Scintillation cocktail, [^3^H] retained on filter was counted using a PerkinElmer TriCarb A4810 liquid scintillation counter.

### Culturing and transfection of T-Rex SERCA2a cells

Stable inducible Flp-In T-Rex-293 cells expressing SERCA2a (44) at 60% to 80% confluency were transiently co-transfected with plasmids containing the cDNAs of GFP-RyR2 and R-CEPIA1er, as previously described (51, 52). After 48 h of expression, cells were used for the confocal experiments to evaluate the changes in the ER luminal [Ca^2+^]([Ca^2+^]_ER)_. Before measurements, cells were permeabilized with 0.005% escin for 3 min. After escin washout, experiments were conducted in a solution of (in mM): K-aspartate 100; KCl 15; KH_2_PO_4_ 5; MgATP 5; EGTA 0.35; CaCl_2_ 0.22; MgCl_2_ 0.75; HEPES 10; dextran (MW: 40,000) 2% and pH 7.2. Free [Ca^2+^] and [Mg^2+^] of this solution were 100 nM and 1 mM, respectively.

### Isolation of ventricular myocytes

Male and female C57Bl6/J mice, (3 animals, Jackson Laboratories) were housed according to approved IACUC guidelines. Mice aged between 2 and 5 months were anesthetized using isoflurane (1%). After thoracotomy, hearts were quickly excised, immersed in Ca^2+^ free buffer, mounted on a Langendorff apparatus, and retrogradely perfused with a solution (37 °C) containing Liberase H (Roche), according to a procedure described previously (72). The left ventricle was excised from the digested heart, placed in stop buffer containing BSA 1 mg/ml, cut into several pieces (average size 1 mm), and gently triturated into single cells. Myocytes were pelleted by gravity (0.1 ml) and resuspended in low-Ca^2+^ Tyrode buffer (140 mM NaCl; 4 mM KCl; 1 mM CaCl_2_; 1 mM MgCl_2_; 10 mM glucose; 10 mM Hepes; pH 7.4). Isolated cardiomyocytes were stored at room temperature (20 °C).

### Confocal microscopy

Changes in the cytosolic [Ca^2+^] ([Ca^2+^]_i_) and the luminal ER [Ca^2+^] ([Ca^2+^]_ER_) were measured using Fluo-4 with laser scanning confocal microscopy, as described previously (52). Ventricular myocytes were incubated at room temperature with 10 μM Fluo-4 AM for 15 min in Tyrode solution (140 mM NaCl; 4 mM KCl; 1 mM CaCl_2_; 1 mM MgCl_2_; 10 mM glucose; 10 mM Hepes; pH 7.4), followed by a 20-min wash. During the experiments, ventricular cardiomyocytes were field stimulated at 0.5 Hz and perfused with 1 mM Ca^2+^ Tyrode. The 5 mM caffeine-induced Ca^2+^ transients were acquired while field stimulation was momentarily paused. The electrical stimulation was resumed after [Ca^2+^]_i_ returned to basal levels. Fluo-4 was excited at 488 nm, and emission signals were acquired above 515 nm. Changes in [Ca^2+^]_i_ were expressed as changes in F/F0, where F0 is the Fluo-4 signal at the resting condition.

[Ca^2+^]_ER_ was recorded as changes in fluorescence intensity of the genetically encoded ER-targeted Ca^2+^ sensor R-CEPIA1er in Flp-In T-Rex-293 cells (51, 73). R-CEPIA1er was excited at 543 nm, and emission was collected at > 580 nm. Every 5 s, 2D images (512 × 512 pixels) were collected at a 6 ms/line scanning speed. The changes in [Ca^2+^]_ER_ were calibrated according to the following formula: [Ca^2+^]_ER_ = (F-F_min_)/(F_max_-F_min_). F_min_ was recorded after ER Ca^2+^ depletion with 10 mM caffeine, and F_max_ was recorded in 10 mM Ca^2+^ and 2 μM ionomycin.

All 2D images and line scan measurements for [Ca^2+^]_SR_ or [Ca^2+^]_ER_ were analyzed with ImageJ software.

### Analysis and presentation of data

[Ca^2+^]_ER_ data are presented as mean ± SD. Groups were compared using paired, two-tailed Student’s *t* test. Statistical analysis and graphical representation of averaged data were carried out on Origin 2021b SR2 software (OriginLab). All other data are presented as mean ± SD. For statistical difference determination, we used unpaired, two-tailed Student’s *t* test. Significance was accepted at *p* < 0.05.

## Data availability

All data are available through the data repository for the University of Minnesota, using this link https://doi.org/10.13020/q0nb-z977.

## Supporting information

The article contains supporting information.

## Conflict of interest

The authors declare the following financial interests/personal relationships which may be considered as potential competing interests: D. D. T. and J. J. T. hold equity in and serve as executive officers for Photonic Pharma LLC. These relationships have been reviewed and managed by the University of Minnesota. The present research is a pre-commercial collaboration between UMN and Photonic Pharma. R. N., E. B., L. A. T., M. B., K. B., A. R. T., C. C. A., A. V. Z., D. M. B., R. L. C. and R. T. R. have no conflicting interests to declare. Razvan Cornea is currently an employee of the National Institutes of Health. This work was conducted during his previous employment, at University of Minnesota – Twin Cities. The opinions expressed in this article are the author's own and do not reflect the view of the National Institutes of Health, the Department of Health and Human Services, or the United States government.
