# Supplementary material for: Large-scale high-throughput screen for cardiac ryanodine receptor targeted therapeutics
Source: J Biol Chem. 2025 Nov 17;302(1):110951. doi: 10.1016/j.jbc.2025.110951 (PMC12765055; doi:10.1016/j.jbc.2025.110951)
Supplement: Supporting information [file mmc1.pdf]

Supporting Information

Large-scale high-throughput screen for cardiac ryanodine receptor targeted therapeutics

Roman Nikolaienko[1†], Elisa Bovo[1†], Jonathan C. Solberg[2†], Marzena Brinkmann[3], Levy M. Treinen[2], Andrew R. Thompson[2], Kaja Berg[3], David D. Thomas[2,4], Jennifer J. Thomas[4], Donald M. Bers[5], Courtney C. Aldrich[3], Aleksey V. Zima[1], Razvan L. Cornea[2], and Robyn T. Rebbeck[2]*

[1]Department of Cell and Molecular Physiology, Loyola University Chicago, Stritch School of Medicine, Maywood, Illinois 60153; USA

[2]Department of Biochemistry, Molecular Biology, and Biophysics, University of Minnesota, Minneapolis, MN 55455, USA;

[3]Department of Medicinal Chemistry, University of Minnesota, Minneapolis, MN 55455, USA;

[4]Photonic Pharma LLC, Minneapolis, MN, 55410, USA.

[5] Department of Pharmacology, University of California at Davis, Davis, CA, USA 95616.

†Co-first author contributions

Supplementary Figures

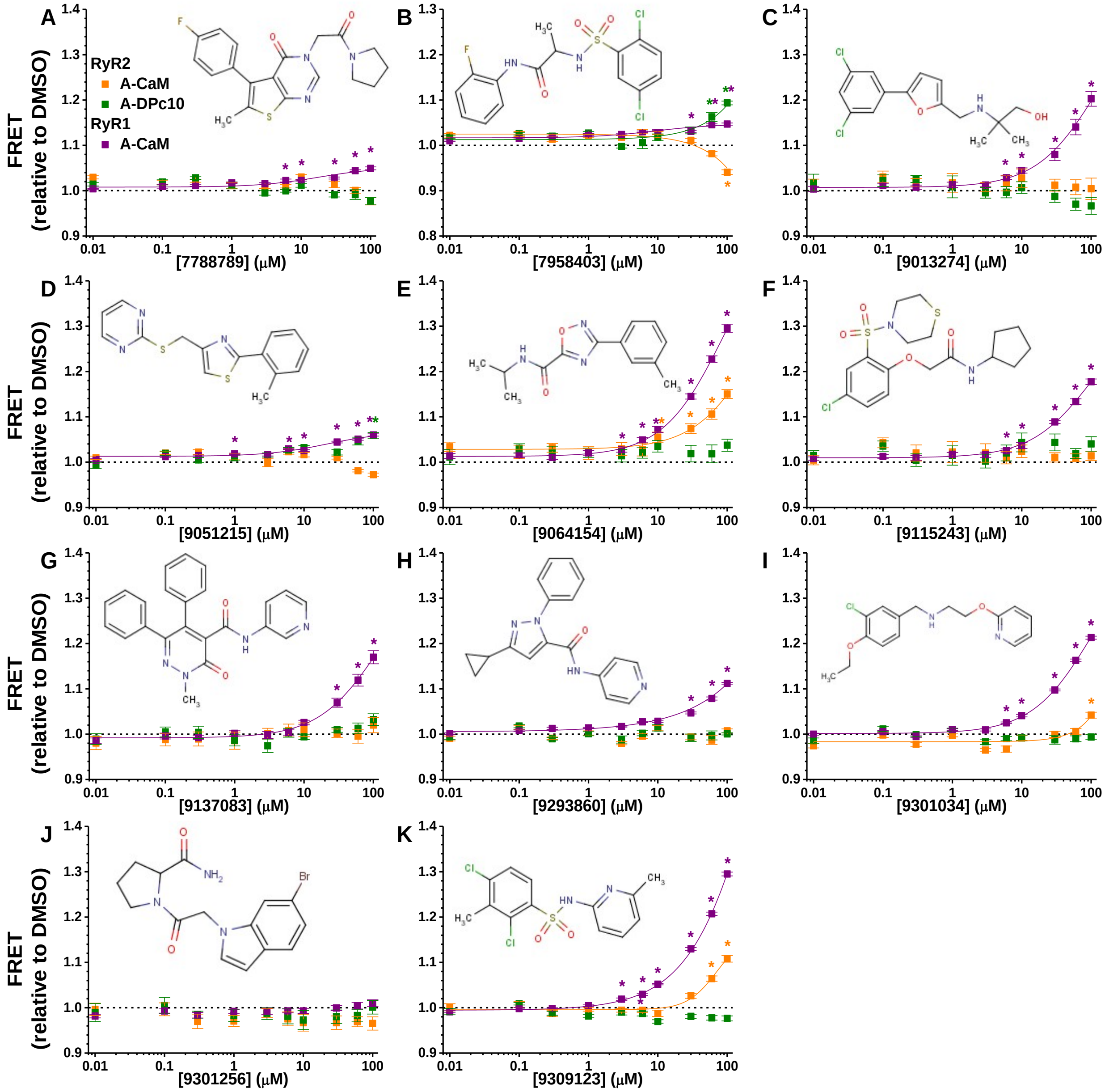

Figure S1 FRET dose-response with structurally unique compounds. The effect of each ChemBridge compound on FRET between D-FKBP and A-CaM on RyR1 (purple), A-CaM on RyR2 (orange) and A-DPc10 on RyR2 (green). Effects shown for ChemBridge compounds 7788789 (A), 7958403 (B), 9013274 (C), 9051215 (D), 9064154 (E), 9115243 (F), 9137083 (G), 9293860 (H), 9301034 (I), 9301256 (J) and 9309123 (K). Data shown as mean±SD, n = 3 individual experiments. *Significance from DMSO control, p <0.05, using unpaired, two-way Student's t-test.

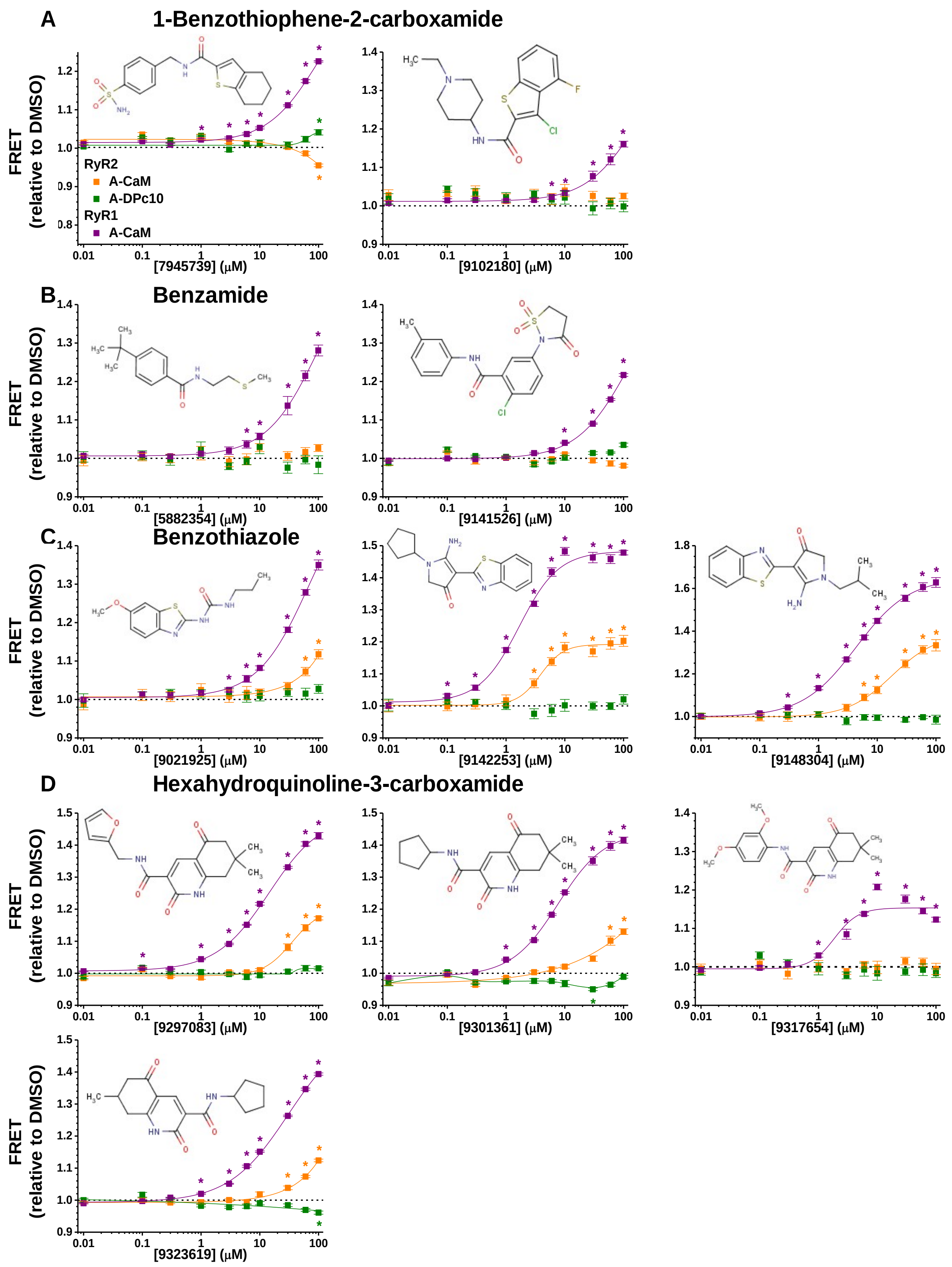

Figure S2 FRET dose-response with chemically clustered groups. The effect of each compound on FRET between D-FKBP and A-CaM on RyR1 (purple), A-CaM on RyR2 (orange) and A-DPc10 on RyR2 (green). FRET dose responses for A) 1-benzothiophene-2-carboxamides, B) benzamides, C) benzothiazoles, and D) hexahydroquinoline-3-carboxamides. Relative to DMSO control, data shown as mean±SD, n = 3 individual experiments. *Significance from DMSO control, $p < 0.05$, using unpaired, two-way Student's t-test.

Indole-3-glyoxamide

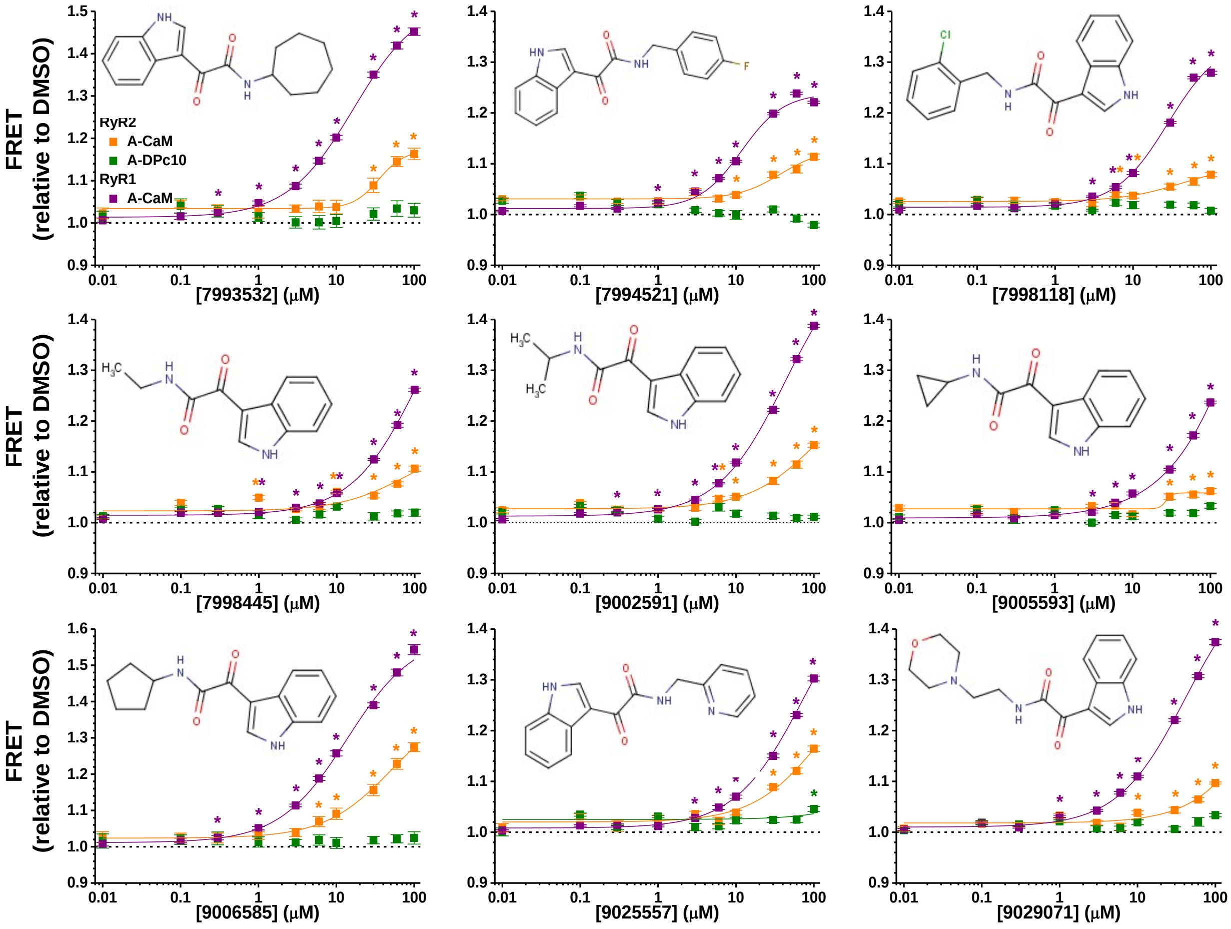

Figure S3 FRET dose-response of Indole-3-glyoxamide compound class. The effect of each compound on FRET between D-FKBP and A-CaM on RyR1 (purple), A-CaM on RyR2 (orange) and A-DPc10 on RyR2 (green). FRET dose responses for indole-3- glyoxamide Hits. Relative to DMSO control, data shown as mean±SD, n = 3 individual experiments. *Significance from DMSO control, $p < 0.05$, using unpaired, two-way Student's t-test.

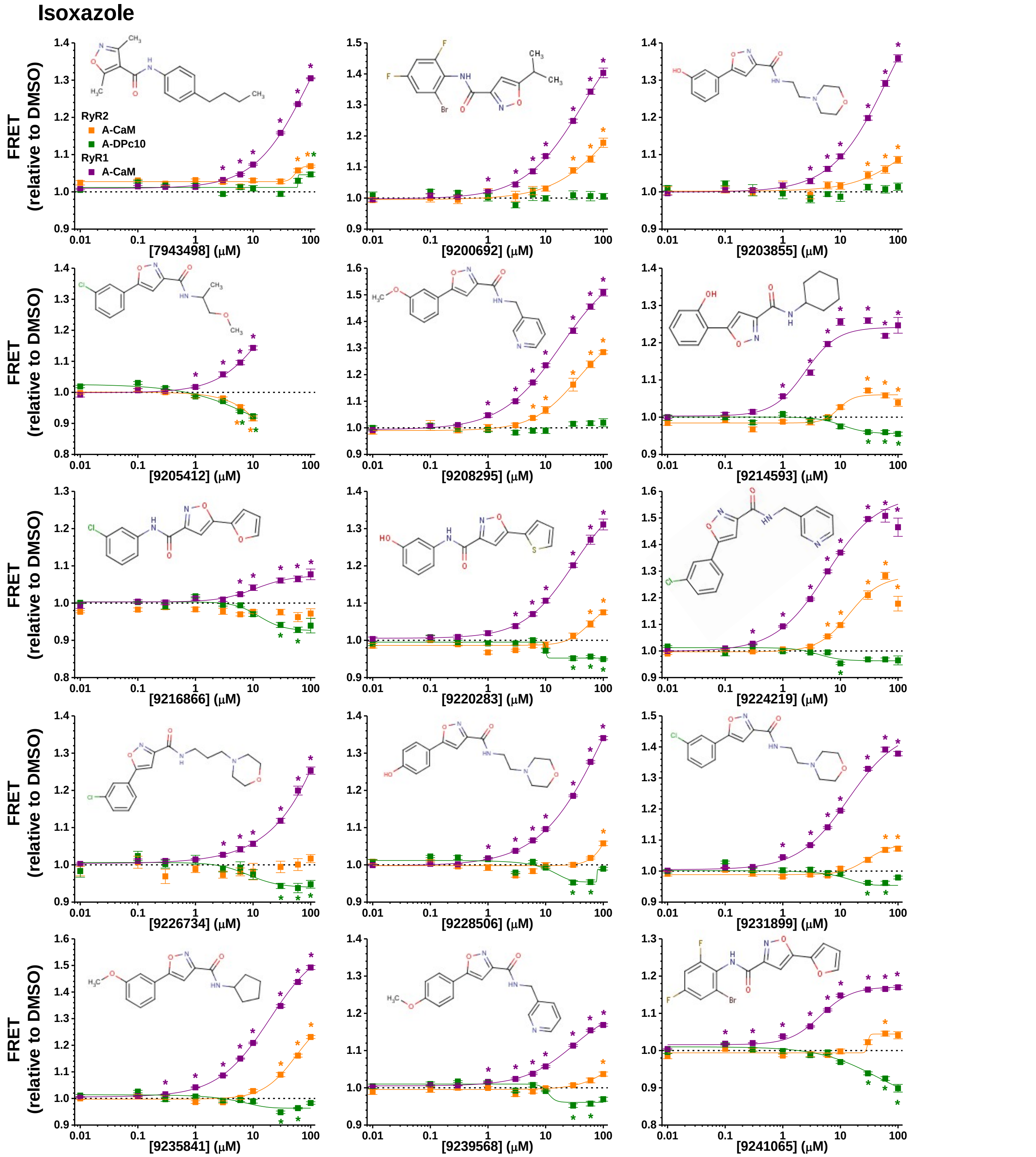

Figure S4 FRET dose-response of Isoxazole compound class. The effect of each compound on FRET between D-FKBP and A-CaM on RyR1 (purple), A-CaM on RyR2 (orange) and A-DPc10 on RyR2 (green). FRET dose responses for isoxazole Hits. Relative to DMSO control, data shown as mean±SD, n = 3 individual experiments. *Significance from DMSO control, $p < 0.05$, using unpaired, two-way Student's t-test.

Isoxazole continued

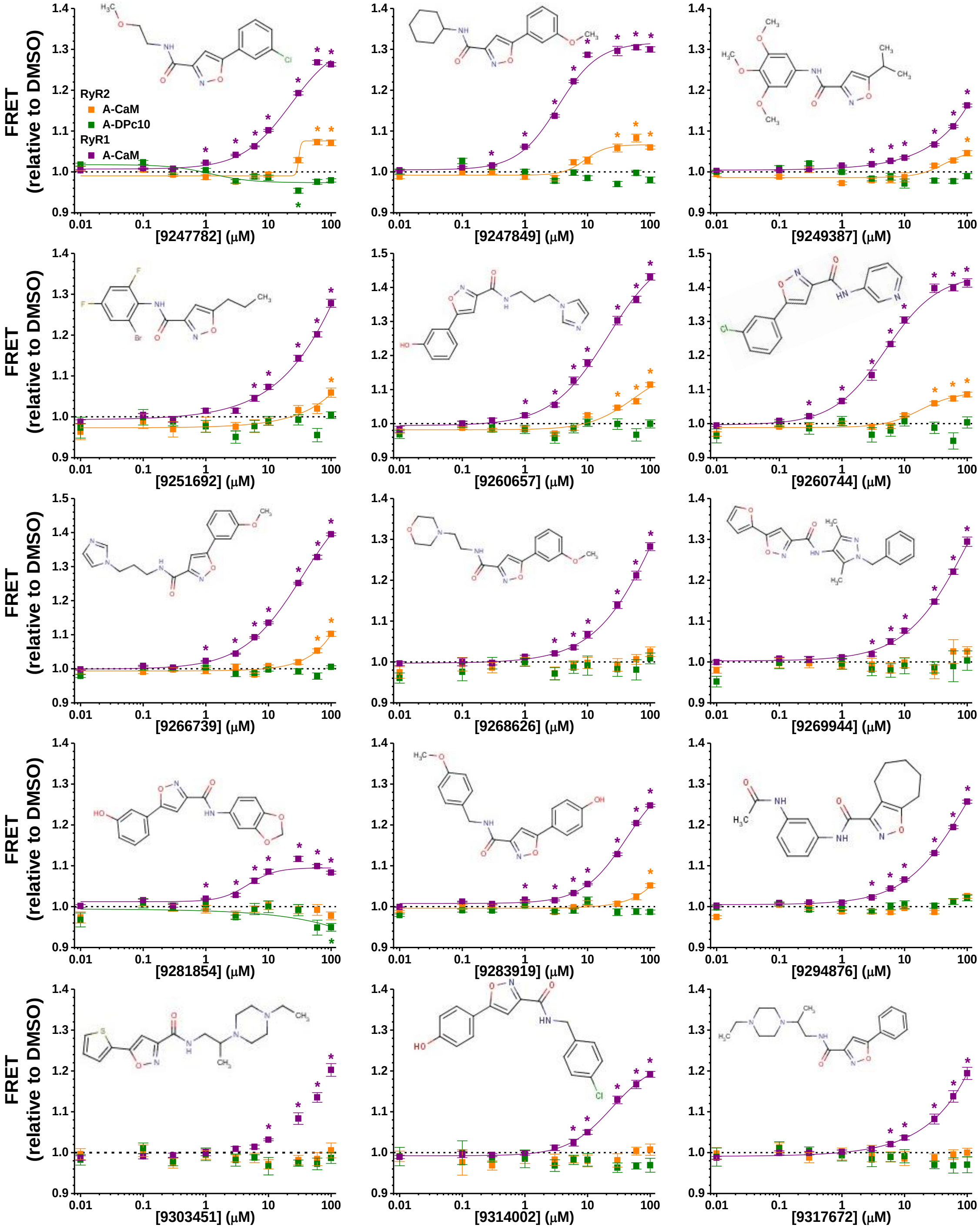

Figure S5 FRET dose-response of Isoxazole compound class. The effect of each compound on FRET between D-FKBP and A-CaM on RyR1 (purple), A-CaM on RyR2 (orange) and A-DPc10 on RyR2 (green). FRET dose responses for isoxazole Hits. Relative to DMSO control, data shown as mean±SD, n = 3 individual experiments. *Significance from DMSO control, $p < 0.05$, using unpaired, two-way Student's t-test.

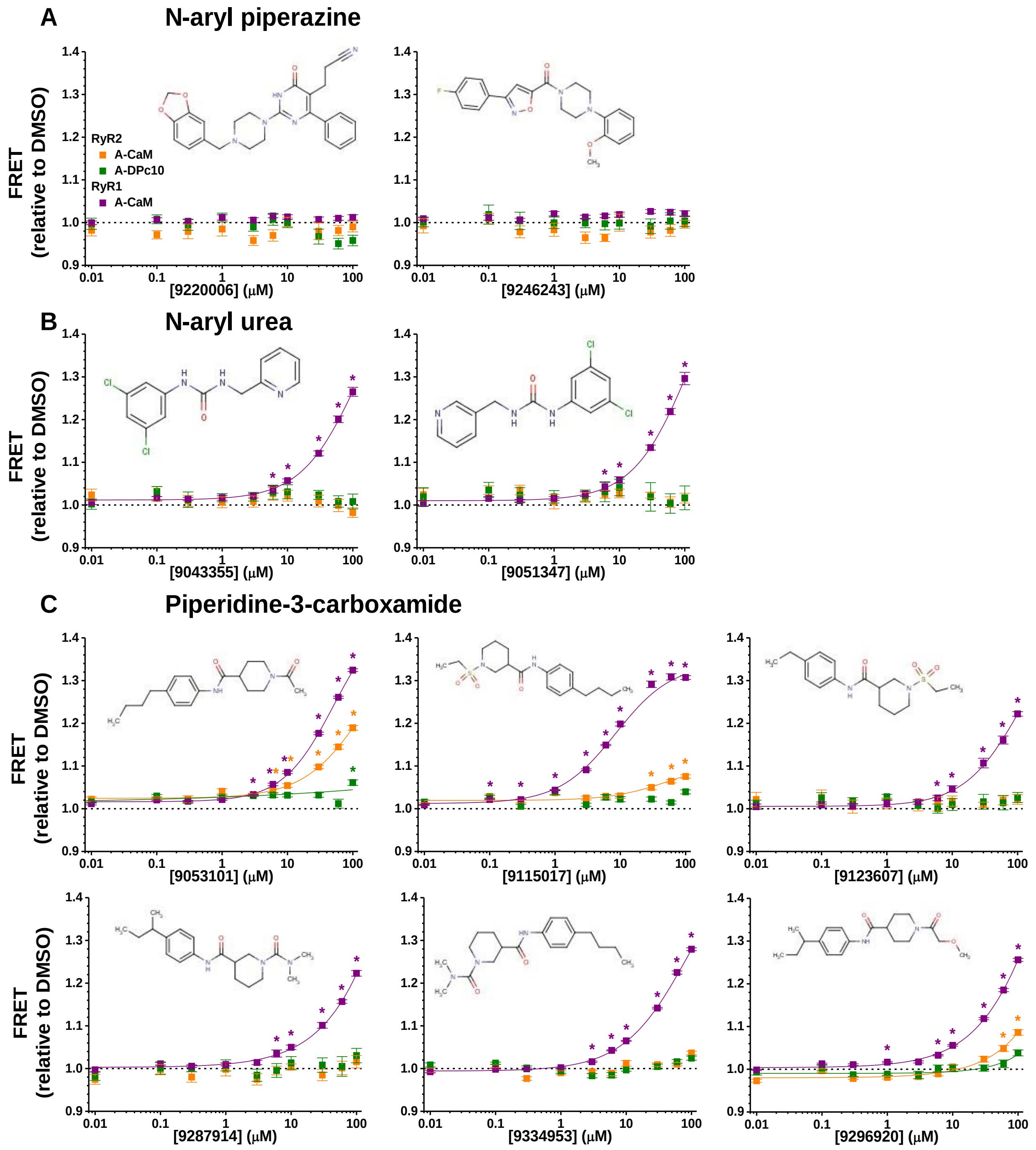

Figure S6 FRET dose-response with chemically clustered groups. The effect of each compound on FRET between D-FKBP and A-CaM on RyR1 (purple), A-CaM on RyR2 (orange) and A-DPc10 on RyR2 (green). FRET dose responses for A) N-arylpiperazine, B) N-aryl urea, and C) piperidine-3-carboxamide Hit compounds. Relative to DMSO control, data shown as mean±SD, n = 3 individual experiments. *Significance from DMSO control, $p < 0.05$, using unpaired, two-way Student's t-test.

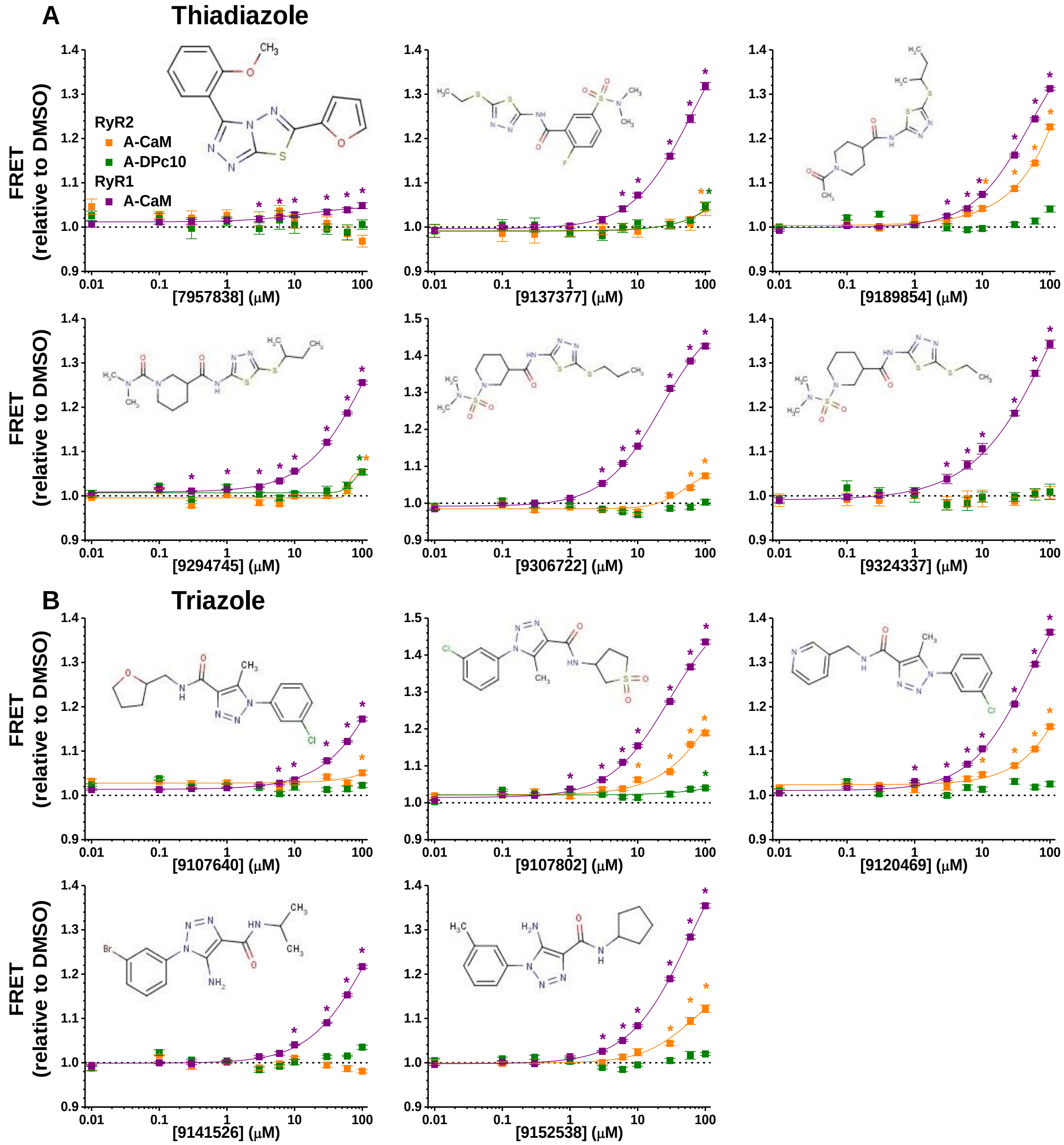

Figure S7 FRET dose-response with chemically clustered groups. The effect of each compound on FRET between D-FKBP and A-CaM on RyR1 (purple), A-CaM on RyR2 (orange) and A-DPc10 on RyR2 (green). FRET dose responses for A) thiadiazole and B) triazole Hit compounds. Relative to DMSO control, data shown as mean±SD, n = 3 individual experiments. *Significance from DMSO control, $p < 0.05$, using unpaired, two-way Student's t-test.

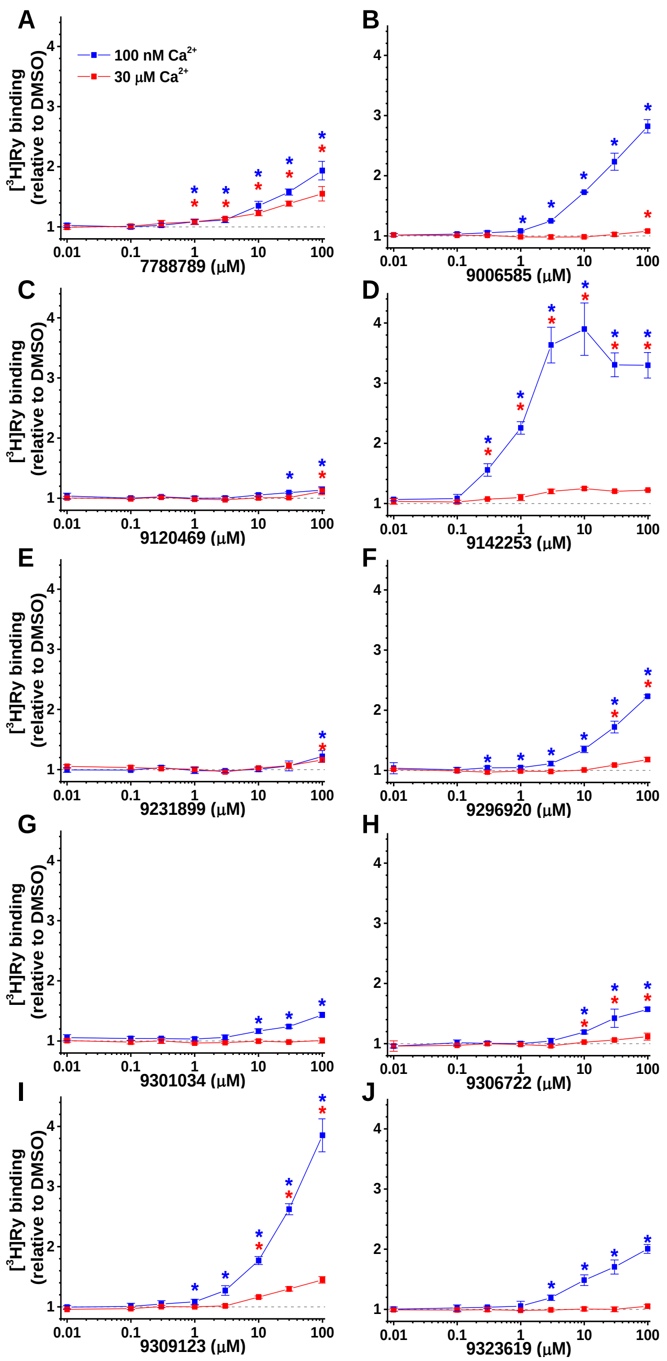

Figure S8 [3H]ryanodine binding profiles for RyR1 in the presence of cluster representative Hit compounds. Dose-dependent (0–100 µM) effect of compounds on [^{3}H]ryanodine binding to cardiac SR (RyR2) at 100 nM (blue) or 30 µM (red) free Ca^{2+}. Dose response representatives of A) chemically unique 7788789, B) indole-3-glyoxamide, C) triazole, D) benzothiazole, E) isoxazole, F) piperidine-3-carboxamide, G) chemically unique 9301034, H) thiadiazole, I) chemically unique 9309123, and J) hexahydroquinoline-3-carboxamide. Data are shown relative to DMSO control, means ± SD, n = 3. *$P < 0.05$ for samples vs. DMSO control using Student's two-way, unpaired t-test.

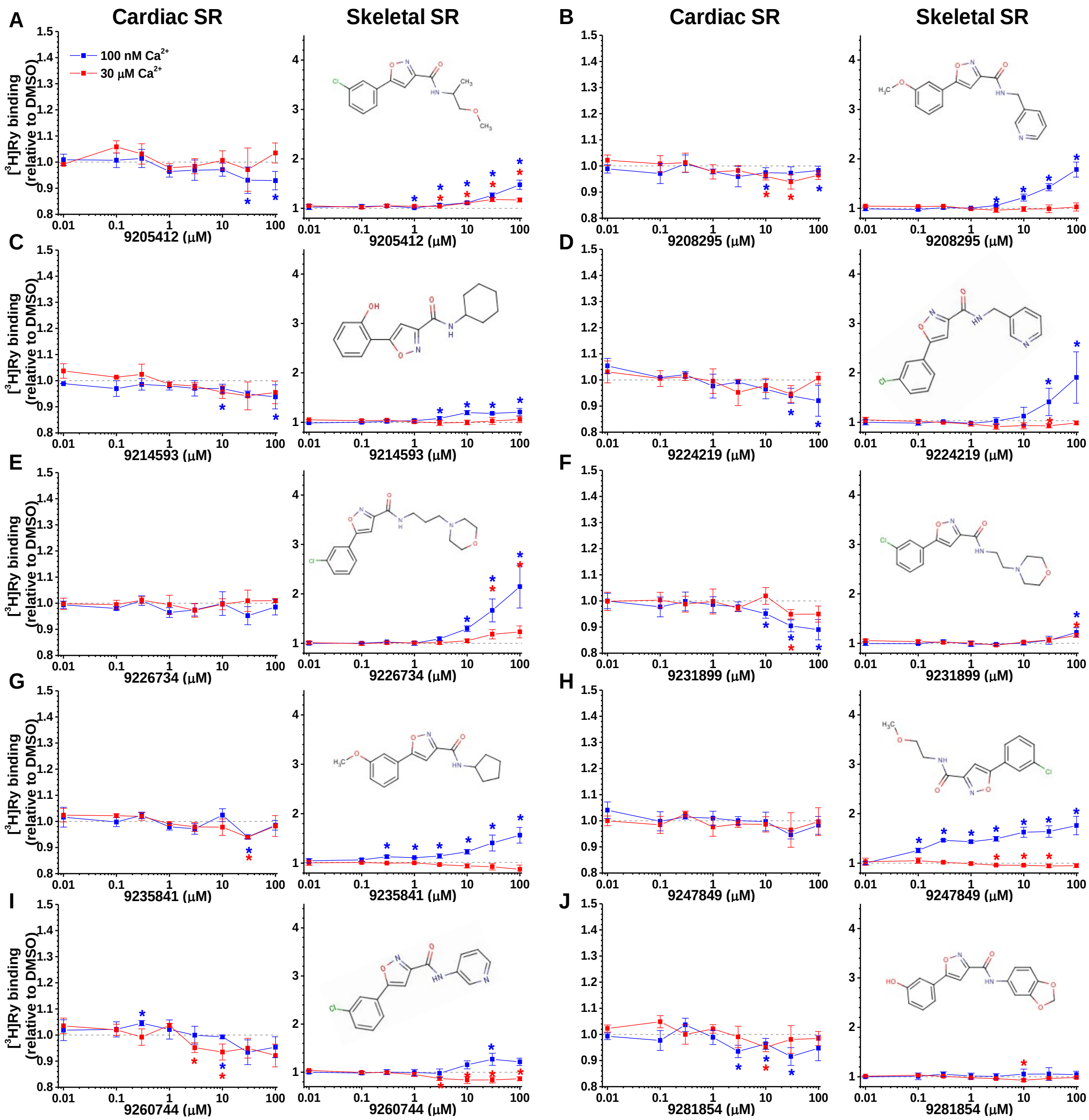

Figure S9 [^{3}H]ryanodine binding profiles for cardiac and skeletal SR in the presence of isoxazole Hit compounds. Dose-dependent (0–100 μM) effect of compounds on [^{3}H]ryanodine binding to cardiac SR (RyR2;left panel) or skeletal SR (RyR2; right panel) at 100 nM (blue) or 30 μM (red) free Ca^{2+}. Dose responses for isoxazole ChemBridge compounds A) 9205412, B) 9208295, C) 9214593, D) 9224219, E) 9226734, F) 9231899, G) 9235841, H) 9247849, I) 9260744, and J) 9281854. Data are shown relative to DMSO control, means ± SD, n = 3. *P < 0.05, for samples vs. DMSO control using two-way, unpaired Student's t-test.
